# The Western Dental Journal

**Published:** 1887-02

**Authors:** 


### THE WESTERN DENTAL JOURNAL.

The initial number of this new aspirant to dental favor has made its appearance, and a good appearance it makes too. The leading original article is by Dr. H. W. Howe, of Lawrence, Kansas, and is entitled "A New Method of Mounting an Artificial Denture." At the last meeting of the Kansas State Dental Society, Dr. Howe demonstrated the making of one of these dentures, from the rolling out of the gold to the final fastening of the teeth with pink rubber.

Upon its completion, the piece was purchased by the society and presented to the editor of this journal as a souvenir of his visit, and among all his professional treasures there are none which he more highly values, for it is as perfect a piece of workmanship as he possesses. He has exhibited it to many dentists, and few have been able to detect the secret of its construction. The article in question tells how to produce such a piece.

The number contains other original articles, with an excellent resumé of current literature, editorials, extracts, and miscellany. $2.00 per annum. Address R. I. Pearson & Co., publishers, Kansas City, Mo.

